# Delayed boosting improves human antigen-specific Ig and B cell responses to the RH5.1/AS01_B_ malaria vaccine

**DOI:** 10.1172/jci.insight.163859

**Published:** 2023-01-24

**Authors:** Carolyn M. Nielsen, Jordan R. Barrett, Christine Davis, Jonathan K. Fallon, Cyndi Goh, Ashlin R. Michell, Catherine Griffin, Andrew Kwok, Carolin Loos, Samuel Darko, Farida Laboune, Mehmet Tekman, Ababacar Diouf, Kazutoyo Miura, Joseph R. Francica, Amy Ransier, Carole A. Long, Sarah E. Silk, Ruth O. Payne, Angela M. Minassian, Douglas A. Lauffenburger, Robert A. Seder, Daniel C. Douek, Galit Alter, Simon J. Draper

**Affiliations:** 1 University of Oxford, Oxford, Oxfordshire, United Kingdom.; 2 Department of Biological Engineering, MIT, Cambridge, Massachusetts, USA.; 3 Ragon Institute of Massachusetts General Hospital (MGH), MIT and Harvard, Boston, Massachusetts, USA.; 4 Wellcome Center for Human Genetics, University of Oxford, Oxford, Oxfordshire, United Kingdom.; 5 Vaccine Research Center, NIAID/NIH, Bethesda, Maryland, USA.; 6 Institute of Experimental and Clinical Pharmacology and Toxicology, Faculty of Medicine, University of Freiburg, Freiburg, Germany.; 7 Laboratory of Malaria and Vector Research, NIAID/NIH, Rockville, Maryland, USA.

**Keywords:** Immunology, Vaccines, Adaptive immunity, Cellular immune response, Immunoglobulins

## Abstract

Modifications to vaccine delivery that increase serum antibody longevity are of great interest for maximizing efficacy. We have previously shown that a delayed fractional (DFx) dosing schedule (0-1-6 month) — using AS01_B_-adjuvanted RH5.1 malaria antigen — substantially improves serum IgG durability as compared with monthly dosing (0-1-2 month; NCT02927145). However, the underlying mechanism and whether there are wider immunological changes with DFx dosing were unclear. Here, PfRH5-specific Ig and B cell responses were analyzed in depth through standardized ELISAs, flow cytometry, systems serology, and single-cell RNA-Seq (scRNA-Seq). Data indicate that DFx dosing increases the magnitude and durability of circulating PfRH5-specific B cells and serum IgG1. At the peak antibody magnitude, DFx dosing was distinguished by a systems serology feature set comprising increased FcRn binding, IgG avidity, and proportion of G2B and G2S2F IgG Fc glycans, alongside decreased IgG3, antibody-dependent complement deposition, and proportion of G1S1F IgG Fc glycan. Concomitantly, scRNA-Seq data show a higher CDR3 percentage of mutation from germline and decreased plasma cell gene expression in circulating PfRH5-specific B cells. Our data, therefore, reveal a profound impact of DFx dosing on the humoral response and suggest plausible mechanisms that could enhance antibody longevity, including improved FcRn binding by serum Ig and a potential shift in the underlying cellular response from circulating short-lived plasma cells to nonperipheral long-lived plasma cells.

## Graphical abstract

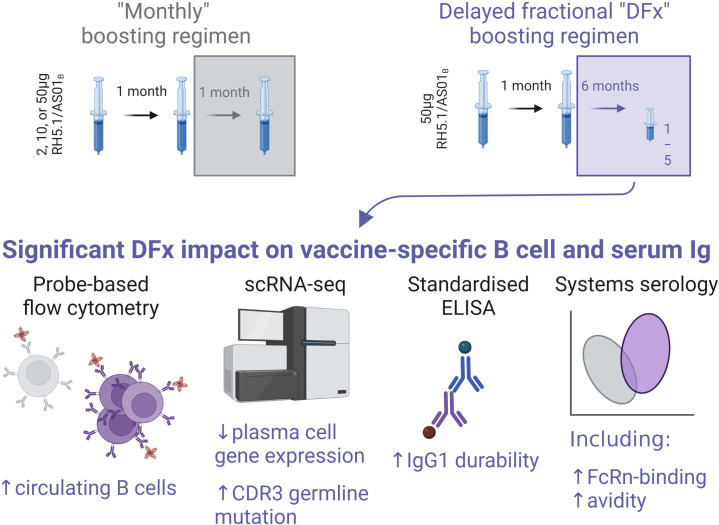

## Introduction

Vaccines are among public health’s most effective tools for combatting infectious disease, but a poor understanding of the underlying immunological mechanisms frequently impedes vaccine development. One of the greatest perennial issues for the vaccinology field is a lack of knowledge regarding how to induce more durable immune responses in the target populations. While many vaccine candidates generate encouraging peak antibody concentrations, these often wane rapidly in the following months. This rapid decay can be highly problematic and poses a particular issue for pathogens such as blood-stage malaria, where the threshold antibody concentration required for protection is high (1, 2).

Interestingly, in a clinical trial with the leading blood-stage malaria vaccine candidate (RH5.1/AS01_B_), we have recently shown that a delayed fractional (DFx) booster 0-1-6 month schedule induces more durable vaccine-specific antibody as compared with a more typically used 0-1-2 month “monthly” vaccination schedule (NCT02927145). Specifically, DFx vaccinee anti-PfRH5 serum IgG peaks in a similar range to monthly vaccinees, but it then plateaus at a 10***×*** higher concentration over the next 2 years — an unprecedented finding (1). Given the strong relationship of anti-PfRH5 antibody concentration with serum in vitro parasite growth inhibition activity (GIA) and in vivo protection (1), such a profound impact in antibody durability is highly relevant to the longevity of vaccine-mediated protection. While data from other malaria vaccine trials — and more recent SARS-CoV-2 trials (3–5) — are broadly supportive of a beneficial impact of delayed (fractional) booster dosing on antibody-mediated immunity, there is yet to be any other demonstration of a comparable impact of vaccine regimen on human antibody longevity or any analyses involving the direct detection and isolation of antigen-specific B cells (6–9).

Here, using samples from the RH5.1/AS01_B_ trial, we have interrogated the PfRH5-specific antibody and B cell responses in both DFx and monthly regimens (Table 1). Through a combination of immunokinetic, systems serology, and single-cell RNA-Seq (scRNA-Seq) analyses, we have identified features of the PfRH5-specific Ig and B cell responses that discriminate between these dosing regimens. These data are informative for understanding the potential underlying mechanisms of DFx-mediated improvements in humoral immunity and will be of great relevance for efforts to further optimize durable antibody responses against malaria and other diseases where vaccine-induced protection is antibody mediated. Specifically, this study focuses on the impact of DFx dosing as compared with monthly booster regimens; further clinical trials will be required to confirm any differential effects of the delayed boosting versus the fractional dose. This work also builds on previously published data demonstrating improved PfRH5-specific IgG and Tfh2 cell immunogenicity following PfRH5 delivery by RH5.1/AS01_B_ as compared with a heterologous viral vector platform (NCT02181088) (1, 10, 11).

## Results

### Delayed fractional (DFx) dosing improves longevity of circulating PfRH5-specific B cells and IgG1.

**Figure 1:**
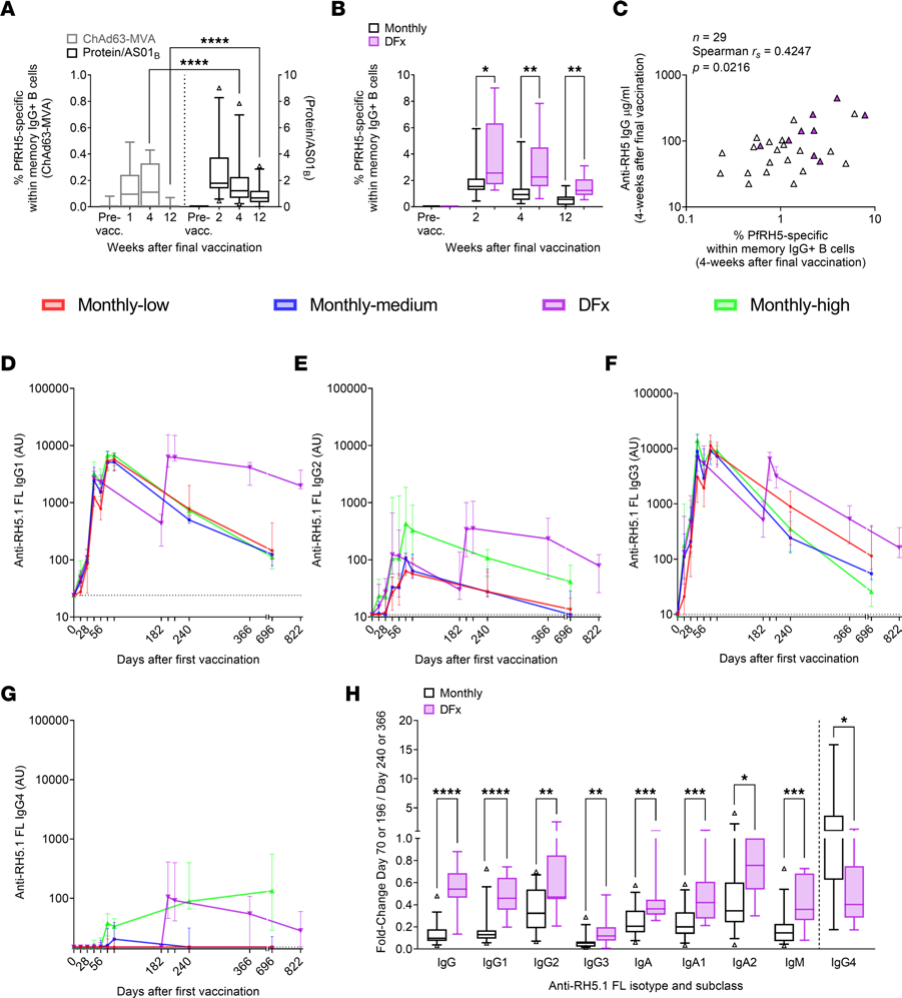
Antigen-specific B cell and Ig postvaccination kinetics in DFx and monthly dosing regimens. PBMC from prevaccination (Pre-vacc.) and post–final vaccination time points were analyzed by flow cytometry. (**A** and **B**) Frequencies of PfRH5-specific mBCs were defined as in Supplemental Figure 1A and compared between heterologous viral vector (ChAd63-MVA; ChAd63-PfRH5 prime, MVA-PfRH5 boost; refs. 10, 11) and protein/AS01_B_ vaccinees (1), or monthly and DFx regimen protein/AS01_B_ vaccinees. (**C**) Spearman’s correlation analysis was performed between PfRH5-specific mBCs and anti-PfRH5 serum IgG (4 weeks after final vaccination); protein/AS01_B_ vaccinees only. Each triangle represents 1 vaccinee; purple triangles indicate DFx vaccinees. (**D**–**G**)Anti-full-length (FL) RH5.1 serum Ig was assayed by standardized ELISA to report IgG1, IgG2, IgG3, and IgG4. (**H**) Fold change in serum anti–PfRH5.1 FL Ig between 2 weeks (Monthly: Day 70; DFx: Day 196) and 6 months after final vaccination (Monthly: Day 240; DFx: Day 366). Full kinetics for IgA, IgA1, IgA2, and IgM, as well as a group-stratified version of **H**, are shown in Supplemental Figure 2. The relationship between anti-RH5.1 IgG and antiparasite functionality is shown in Supplemental Figure 3. Sample sizes for all assays were based on sample availability. (**A**) ChAd63-MVA/protein/AS01_B_: Pre-vacc. *n* = 15/18; 1-week *n* = 10/0; 2-week *n* =0/25; 4-week *n* =15/29; 12-week *n* = 13/25. (**B**) Monthly/DFx: Pre-vacc. *n* = 15/3; 2-week *n* = 16/9; 4-week *n* = 19/10; 12-week *n* = 17/8. (**D**–**G**) Monthly-low: *n* =12, except Day 696 (*n* = 9). Monthly-medium: *n* = 12, except Day 240 (*n* = 11) and Day 696 (*n* = 10). DFx: *n* = 12, except Day 366 (*n* = 11) and Day 822 (*n*=7). Monthly-high: *n* = 11, except Day 70 (*n* = 9), Day 240 (*n* = 10), and Day 696 (*n* = 4). (**H**) Monthly/DFx: *n* = 31/11 except IgG4 (*n* = 18/9). (**A**, **B**, and **H**) Comparisons were performed between regimens with Mann-Whitney *U* tests. Central box lines indicate medians, and whiskers denote 5th and 95th percentiles; samples outside the 5th–95th percentile range are shown as triangles. *P < 0.05, **P < 0.01, ***P < 0.001, ****P < 0.0001.

Using PfRH5 probes to detect circulating PfRH5-specific memory IgG^+^ B cells (defined as live CD19^+^CD21^+^CD27^+^IgG^+^probe^++^ single lymphocytes; Supplemental Figure 1A; supplemental material available online with this article; https://doi.org/10.1172/jci.insight.163859DS1), we first established that protein/AS01_B_ vaccination induced higher frequencies of these antigen-specific cells at both 4 weeks and 12 weeks after final vaccination, as compared with heterologous viral vectors (Figure 1A and Supplemental Figure 1B). Within the protein/AS01_B_ trial, vaccinees receiving a DFx regimen (Table 1) — rather than the monthly dosing regimen — showed higher responses 2, 4, and 12 weeks after the final vaccination (Figure 1B and Supplemental Figure 1C). PfRH5-specific memory IgM^+^ responses were detectable but minimal (Supplemental Figure 1D). While the source of circulating anti-PfRH5 serum IgG is presumably BM-resident long-lived plasma cells (LLPCs), serum IgG and memory B cells (mBCs) do correlate 4 weeks after the final vaccination (Figure 1C) — suggesting a more robust B cell response across multiple germinal center B cell fate lineages (i.e., mBC and LLPCs) with the DFx regimen.

To delve further into the differences in anti–PfRH5 Ig immunokinetics between DFx and monthly vaccinees, we developed standardized ELISAs for anti–PfRH5 IgG1–4, IgA, IgA1–2, and IgM to assay sera samples from key postvaccination time points (Figure 1, D–G, and Supplemental Figure 2, A–D). Although there was no significant difference at the peak time points (2 or 4 weeks following final vaccination), 6 months after the final vaccination, the median IgG1 AU was 4,130 for DFx vaccinees as compared with 628 AU in the monthly regimen vaccinees (5-fold difference; *P* < 0.0001; Mann-Whitney *U* test). After > 1.5years, the IgG1 median AU values were 1,984 and 133, respectively (15-fold difference; *P* < 0.0001; Mann Whitney *U* test). In the absence of such stark differences in IgG2 (Figure 1E), IgG3 (Figure 1F), and IgG4 (Figure 1G), these data suggest that the majority of the improvement in longevity observed in the total anti–PfRH5 IgG response (1) is attributable to the IgG1 component. Nevertheless, we next calculated the fold change for each of the isotypes and subclasses between the peak response 2 weeks after final vaccination (Day 70 or Day 196) and the 6-month time point (Day 240 or Day 366; Figure 1H). This clearly indicated the slower decay in total IgG (as reported previously; ref. 1) and IgG1 in the DFx vaccinees, but it also showed a significant difference in all other isotypes and subclasses between DFx vaccinees and (pooled) monthly regimen vaccinees. This is an interesting indication that, while DFx may not increase the peak magnitude of these responses, there appears to be a general improvement in serum antibody response maintenance. Of note, IgG4 was the only isotype or subclass measured where decay was significantly faster with the DFx regimen — a signal that was largely driven by the unusual kinetics in the monthly-high vaccinees (Figure 1G and Supplemental Figure 2E).

To confirm the relevance of this durable serum anti–PfRH5 IgG for antiparasitic functionality, we next performed a GIA assay with purified total serum IgG from 6 months after the final vaccination. In vitro GIA demonstrates the capacity of vaccine-induced IgG to block blood-stage malaria parasite invasion of RBCs and is a strong correlate of in vivo protection (1). Here, we observed significantly higher GIA with serum from DFx as compared with monthly-medium vaccinees (Supplemental Figure 3A) and a strong correlation between GIA and total anti-RH5.1 IgG (Supplemental Figure 3B). This is in contrast to our previously published GIA data with samples from the peak of the humoral response — when serum concentrations of anti–PfRH5 IgG are equivalent and where we observed no significant differences in GIA (1).

### Systems serology feature set associated with DFx dosing includes increased FcRn binding, IgG avidity, and proportion of bigalactosylated IgG Fc glycans.

Our stratified antibody isotype and subclass immunokinetic data suggest a significant quantitative impact of DFx dosing on humoral immunogenicity. We therefore next extended these analyses using a systems serology pipeline to integrate qualitative data on Fc biophysical and functional characteristics, measuring a total of 49 parameters (12, 13). As previously described (1), in addition to quantification of postvaccination plasma levels of PfRH5-specific antibodies of each major isotype and subclass (with a Luminex bead–based assay rather than ELISA, as above), this approach extends the biophysical analyses to include characterization of the glycosylation profile of the anti–PfRH5 IgG Fc domains, known to influence Fc-mediated functions (14). To assess Fc-mediated functionality of PfRH5-specific antibodies, the systems serology platform incorporates evaluation of their capacity to bind Fc receptors (FcRs) and to activate monocytes, neutrophils, and NK cells as well as the complement cascade.

**Figure 2:**
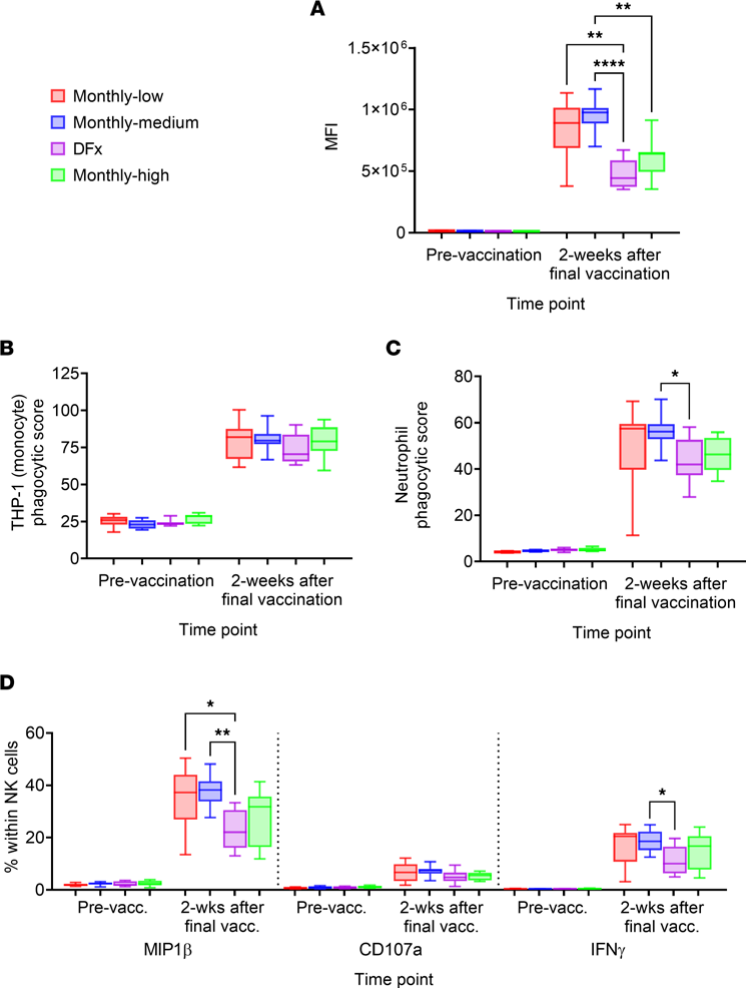
Fc-mediated functionality of peak postvaccination antigen-specific Ig in DFx and monthly dosing regimens. Plasma from prevaccination (Pre-vacc.) and 2 weeks following the final vaccination was assessed for the capacity of anti–PfRH5 Ig to induce Fc-mediated innate immune activation following incubation with PfRH5-coupled beads. (**A**–**D**) The Fc-mediated functionality was compared between dosing regimen with respect to antibody-dependent complement deposition (ADCD), antibody-dependent neutrophil phagocytosis (ADNP), NK cell activation, and antibody-dependent cellular (monocyte) phagocytosis (ADCP). (**A**) Beads were incubated with plasma, and guinea pig complement and C3 complement deposition was detected by staining with an anti-C3 fluorescent antibody and reported with the median anti-C3 fluorescence intensity (MFI) of each sample. (**B**) The functional capacity of pre-/postvaccination plasma to induce antibody-dependent monocyte phagocytosis was compared based on the capacity of anti-PfRH5–bound beads to induce phagocytosis by the THP-1 (monocyte) cell line in the presence of plasma. Phagocytic score of each sample = (% bead^+^ cells) ***×*** (MFI)/(10***×*** MFI of first bead^+^ peak). (**C**) Neutrophils were isolated from fresh blood and then incubated with plasma and beads; they were then stained to define neutrophils as SSC^hi^CD66b^+^CD14^–^CD3^–^ cells. Induction of phagocytosis was compared by calculating phagocytic scores as (% bead^+^ cells) ***×*** (geometric median fluorescence intensity [MFI] of bead-positive cells)/(10 ***×*** gMFI of the first bead^+^ peak). (**D**) NK cells were purified from buffy coats then incubated with plasma and antigen-coated ELISA plates; they were then stained to define NK cells as CD56^+^CD3^–^ cells. Activation was measured as the percentage of NK cells expressing MIP1β, CD107a, or IFN-γ as detected by fluorescent antibodies. Plasma was available from all vaccinees for inclusion in these analyses in technical duplicates. Prevaccination/postvaccination: Monthly-low *n* = 12/12; monthly-medium *n* = 11/11; DFx *n* = 11/11; monthly-high *n* = 9/9. Comparisons between groups were performed by Kruskal-Wallis test with Dunn’s correction for multiple comparisons. Central box lines indicate medians and whiskers denote 5th and 95th percentiles. *P < 0.05, **P < 0.01, ****P < 0.0001.

In initial univariate analyses with the functional read-outs, we observed regimen-dependent differences in Fc-mediated activation with intriguing trends toward reduced functionality in DFx as compared with the monthly-low or monthly-medium vaccinees (Figure 2). Specifically, PfRH5-specific Ig in plasma samples 2 weeks following the final vaccination initiated decreased antibody-dependent complement deposition (ADCD; Figure 2A), antibody-dependent neutrophil phagocytosis (ADNP; Figure 2C), and NK cell cytokine production (MIP1β or IFN-γ; Figure 2D). No differences were observed in NK cell degranulation, as measured by CD107a expression (Figure 2D) or antibody-dependent cellular (monocyte) phagocytosis (ADCP; Figure 2B). It was of interest to note that, in each instance, samples from monthly-high vaccinees performed comparably with DFx vaccinees. This suggested that there was possibly an impact of the “high” 50 μg first and second doses (shared by these 2 groups) on Fc-mediated functionality, independent from any effect of a DFx final booster.

**Figure 3:**
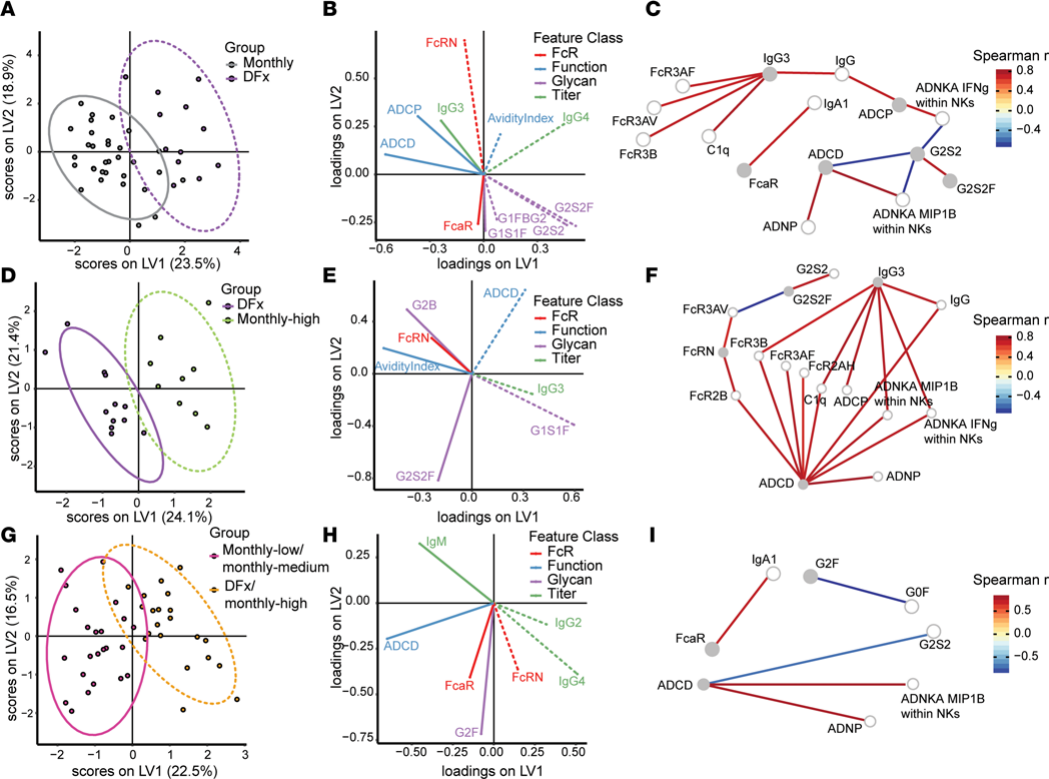
Systems serology computational analyses to define Ig feature sets that distinguish DFx from monthly dosing regimen. Partial least squares discriminant analysis (PLS-DA) was performed with univariate read-outs from the systems serology analyses to compare DFx and monthly dosing regimens. (**A** and **B**) Significant features were chosen via the LASSO feature selection algorithm and those chosen in at least 80% of 100 repetitions were used to build PLS-DA classifiers. Correlation networks were built to reveal additional serology features significantly associated with the selected features. (**C**) Serology features significantly (*P* < 0.05, after a Benjamini-Hochberg correction) correlated via Spearman’s correlation (*r_S_* > |0.7|) were selected as cocorrelates. The gradient color of edges represents correlation value between the features, represented as nodes. Nodes are colored according to selected status, with gray nodes as selected features and white nodes as cocorrelate features. (**D–I**) This approach was also used to directly compare DFx vaccinees with monthly-high and DFx/monthly-high vaccinees with monthly-low/monthly-medium regimens. Line style (solid versus hash) of features in **B**, **E**, and **H** relates to group with significant increase in that feature with 95% CI. Models had cross-validation accuracies of 0.85 (**A**), 0.74 (**D**), and 0.83 (**G**), with comparisons to null models generated by random feature selection (*P* = 0.01, *P* = 0.09, and *P* = 0.01, respectively) or permuted labels (*P* < 0.01, *P* = 0.04, and *P* < 0.01, respectively) being significant for all but comparison to a null model built with randomly selected features in DFx versus monthly-high vaccinees. This is likely due to the limited number of samples and the high correlations between selected features and nonselected features (**F**). LV, latent variable; ADCD, antibody-dependent complement deposition; ADCP, antibody-dependent cellular (monocyte THP-1) phagocytosis; ADNP, antibody-dependent neutrophil phagocytosis; ADNKA, antibody-dependent NK cell activation. Monthly-low, *n* = 12; monthly-medium, *n* = 11; DFx, *n* = 12; monthly-high, *n* = 9.

To deconvolute these data, we took 3 approaches with our subsequent computational analyses of the complete systems serology data sets. First, we focused on our original research question by comparing all monthly versus DFx regimens (Figure 3, A–C). Next, we limited this analysis to a direct comparison between the DFx and monthly-high vaccinees; these differ only in their final vaccination and are, thus, optimal comparators (DFx versus monthly-high; Figure 3, D–F). Finally, we addressed the hypothesis raised by the univariate Fc functional data and compared the monthly-low and monthly-medium regimens to the monthly-high and DFx regimens (Figure 3, G–I). The computational pipeline in each instance consisted of performing a partial least squares discriminant analysis (PLS-DA) using features selected via least absolute shrinkage and selection operator (LASSO; Figure 3, A, B, D, E, G, and H), followed by Spearman’s correlation networks to reveal additional serology features significantly associated with the selected features (Figure 3, C, F, and I). Parallel computational analyses correcting for total anti–RH5 IgG yielded comparable results, as expected, given comparable anti-PfRH5 concentrations at the 2-week postvaccination time point (1). It is also important to emphasize that these analyses are defining feature sets that discriminate between groups, rather than univariate read-outs as per (Figure 2). Consequently, feature sets may contain parameters that are not significantly different between groups when analyzed alone (e.g., ADCD; Figure 2A).

The first analysis indicates a feature set able to discriminate between DFx and monthly regimens: FcRn binding, IgG avidity, proportion of 3 different Fc glycans (G1FBG2, G2S2, G2S2F), IgG4 (all higher in DFx vaccinees), IgG3, FcαR binding, ADCD, ADCP, and G1S1F glycan (all higher in monthly regimen vaccinees; Figure 3B and Supplemental Figure 4). The cocorrelate network additionally detects a significant negative association between G2S2 and both NK cell (MIP1β and IFN-γ) and complement cascade activation (Figure 3C). ADCD and ADCP are associated with the monthly regimens, as suggested by the univariate data (Figure 2).

With respect to the second analysis, direct comparison of the DFx regimen with only the monthly-high regimen showed that increased FcRn binding, IgG avidity, G2S2F and G2B glycans, alongside decreased IgG3, ADCD, and G1S1F glycan, were able to discriminate DFx vaccinees (Figure 3E and Supplemental Figure 4). The corresponding cocorrelate network also identified positive correlations between FcRn binding, and FcR2B or FcR3AVbinding, as well as correlations between G2S2F and G2S2 (positive) and FcR3AV binding (negative).

Finally, the computational analyses identified increased FcRn binding, IgG2, and IgG4, in addition to decreased IgM, ADCD, FcαR binding, and G2F glycan as the significant feature set to discriminate the “high” dose vaccinees (Figure 3H). The FcRn binding signature in this analysis is attributable only to the DFx samples: FcRn binding levels separate DFx versus monthly-high (Figure 3E), and removing FcRn binding from the feature set — considered when separating lower versus higher dose groups — does not impact separation (data not shown). The selected features truly elevated in “high” dosing are, accordingly, IgG2 and IgG4. This is consistent with the divergent IgG subclass immunokinetics analyzed by ELISA described above (Figure 1, D–G). No further correlations were identified in the cocorrelates model with any of the 3 parameters elevated in the “high” dose groups (Figure 3I).

### scRNA-Seq 2 weeks following final vaccination indicates a higher proportion of plasma cells in monthly boosting regimen antigen-specific B cell population as compared with DFx dosing regimen.

**Figure 4:**
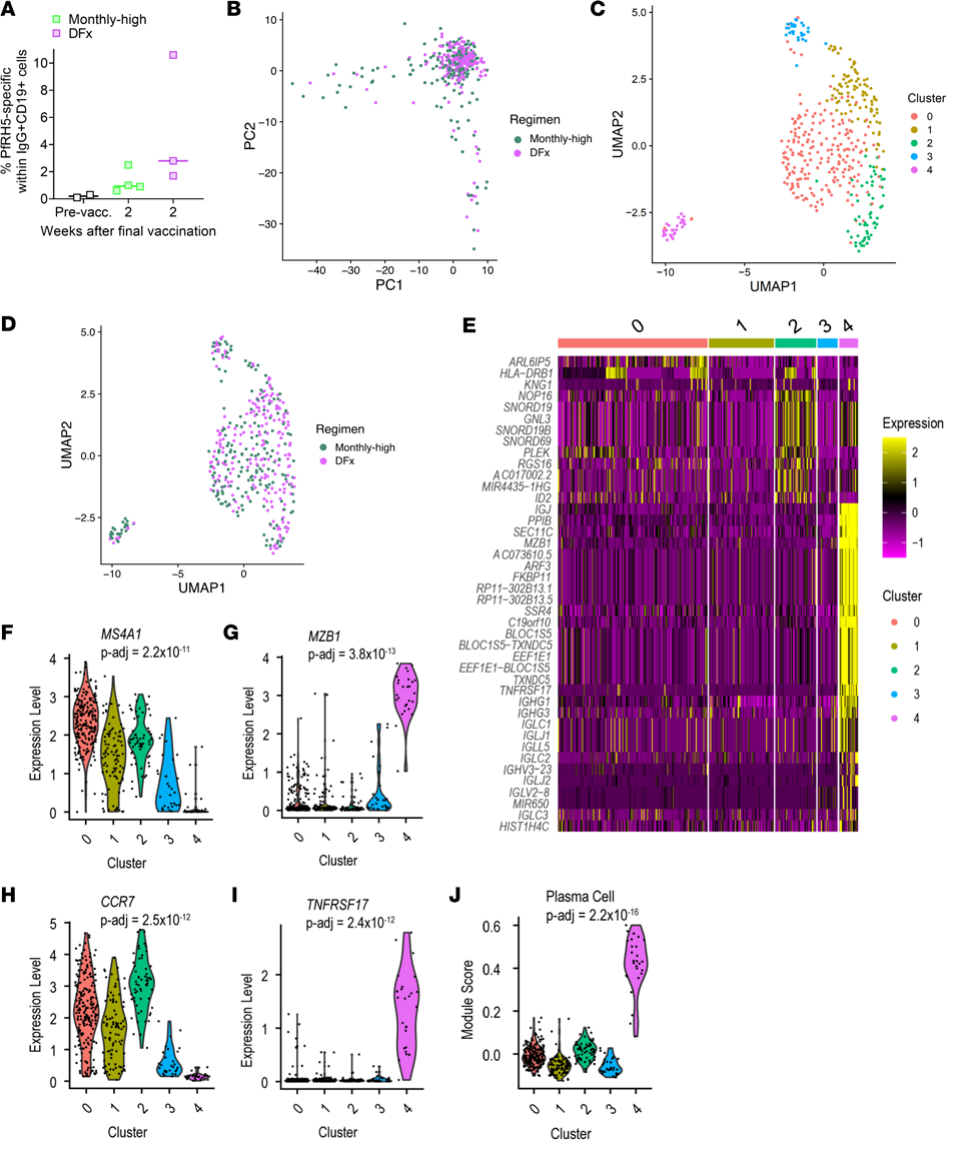
Single-cell RNA-Seq of antigen-specific B cells from DFx and monthly-high dosing vaccinees. PBMC from prevaccination (Pre-vacc.), and 2 weeks after final vaccination in DFx vaccinees (*n* = 3) and monthly-high vaccinees (*n* = 4) were enriched for B cells and then stained with phenotypic markers for single-cell sorting of antigen-specific B cells as defined as: live CD19^+^IgG^+^ lymphocytes that costained for monobiotinylated PfRH5-PE and monobiotinylated PfRH5-APC (gating strategy shown in Supplemental Figure 1A). (**A**) Frequencies of postvaccination PfRH5-specific B cells from the samples sorted were comparable with previous data. Libraries were sequenced following a Smart-Seq v4 and Nextera XT pipeline on a HiSeq4000. (**B**–**E**) Variation in gene expression within the 7 samples was explored in Seurat by PCA analysis of DFx as compared with monthly-high regimen (**B**), UMAP with 5 clusters (**C**), UMAP with 5 clusters with dosing regimen identity overlaid (**D**), and heatmap of the top 50 most differentially expressed genes by cluster (**E**). Expression of genes of interest identified were then compared between cluster 4 and the other clusters by Wilcoxon rank sum test with Bonferroni correction to give adjusted *P* values (*P*_adj_): MS4A1 (CD20) (**F**), MZB1 (**G**), CCR7 (**H**), and TNFRSF17 (**I**). (**J**) Expression of a plasma cell gene set (“TARTE_PLASMA_CELLS_VS_B_LYMPHOCYTE_UP”; ref. 30) was also compared between cluster 4 and the other clusters by Kruskal-Wallis test.

While we had already shown that the DFx regimen induces a higher frequency of circulating CD19^+^IgG^+^ PfRH5-specific B cells (memory cells as shown in Figure 1B), it was not clear whether there were any qualitative differences within this population that might relate to differences in the humoral response described above. To address this, we performed scRNA-Seq with CD19^+^IgG^+^ PfRH5-specific B cells from *n* = 4 monthly-high vaccinees and *n* = 3 DFx vaccinees. The range of frequencies of PfRH5-specific B cells within the live, CD19^+^IgG^+^ population acquired during sorting were comparable with those previously observed (monthly-high regimen 0.6%–2.5%, DFx regimen 1.7%–10.6%; Figure 1B and Figure 4A).

We first explored the heterogeneity in gene expression across all vaccinees within Seurat (Figure 4, B–J). A total of 209 cells from monthly-high vaccinees and 214 cells from DFx vaccinees were included in this analysis. Following normalization of data, we identified the 10 genes with the highest variance across the entire sample set: MZB1, KNG1, LEO1, EAF2, UBXN8, P2RY12, ZNF234, MRPL35, TNFRSF17, IGKV1-39. Two of these genes are noteworthy for their associations with plasma cells that could be highly relevant for understanding our humoral data set: MZB1 and TNFRSF17. MZB1 — also known as plasma cell–induced ER protein (pERp1) — is a key effector of the transcription factor B that regulates terminal plasma cell differentiation (Blimp1) (15–22). TNFRSF17 — also known as BCMA — is the APRIL and BAFF receptor that is restricted to mature B cells and plasma cells (including both short-lived and long-lived) (23–25).

Next, we ran a PCA to identify the number of principal components appropriate for downstream clustering and visualization with Uniform Manifold Approximation and Projection (UMAP) (Figure 4B). Running UMAP on these principal components, we observe phenotypically distinct populations of cells (Figure 4C), one of which appeared to be enriched for monthly-high vaccinee cells (cluster 4; Figure 4, C and D; top 5 genes for each shown in Supplemental Table 1). A heatmap of the top 50 most differentially expressed genes between the clusters highlights increased expression in cluster 4 of multiple Ig genes and multiple genes with functions related to the secretory pathway or protein production and trafficking (e.g., PPIB, ARF3, FKBP11, SEC11C, SSR4, BLOC1S5, and TXNDC5; Figure 4E). To further probe this potential cluster 4 plasma cell phenotype, we compared several genes that should have clear positive or negative expression in a plasma cell population: MS4A1 (CD20), CCR7, MZB1, and TNFRSF17. CD20 and CCR7 are both downregulated during plasma cell differentiation (18, 26–29) and, indeed, here we saw decreased expression in cluster 4 as compared with clusters 0–3 (Figure 4, F and H). Conversely, both MZB1 and TNFRSF17 are expressed almost exclusively in cluster 4 (Figure 4, G and I). Finally, scoring each of the clusters based on expression of a previously identified set of genes upregulated in plasma cells as compared with other B cells (“TARTE_PLASMA_CELLS_VS_B_LYMPHOCYTE_UP”; ref. 30) also yields a strong statistically significant difference between cluster 4 and the other clusters (Figure 4J).

We next proceeded to differential gene expression analyses between monthly-high and DFx regimen PfRH5-specific cells to confirm the indication from the Seurat analysis that there was a discrepancy in the proportion of plasma cells (cluster 4) between dosing regimens. In total, 5,115 genes were differentially expressed (adjusted p value [*P*_adj_] < 0.05; top 30 shown in Table 2). Genes with increased expression in cells from monthly-high as compared with DFx vaccinees include MZB1, IGJ (also known as J Chain and associated with plasma cells; ref. 31), HLA-DQA2, and HLA-DRA. Of the other 25 genes, 17 were immunoglobulin heavy or light chain, likely indicating increased Ig production. Indeed, in a recent single-cell atlas characterization of human tonsillar B cells, MZB1, IGJ and IGH genes were all expressed most highly in the plasmablast population (32). A gene set enrichment analysis (GSEA) with the plasma cell gene set — here run on all significantly differentially expressed genes rather than just cluster 4 — showed a significant enrichment in monthly-high vaccinees (*P* = 0.001; normalized enrichment score [NES] = 2.24; Table 3).

Further exploration of the significantly differentially expressed genes by GSEA with the KEGG mSigDB gene sets indicated significant enrichment (*P*_adj_ < 0.05) in 10 pathways, including: cell adhesion molecules, protein export, and intestinal immune network for IgA production (Supplemental Table 2). Protein secretion and mTORC1 signaling (also related to protein secretion) were similarly flagged during Hallmark GSEA, but no pathway reached statistical significance with an adjusted *P* value (Supplemental Table 3).

### PfRH5-specific B cells show greater CDR3 somatic hypermutation in DFx vaccinees as compared with monthly regimen vaccines.

**Figure 5:**
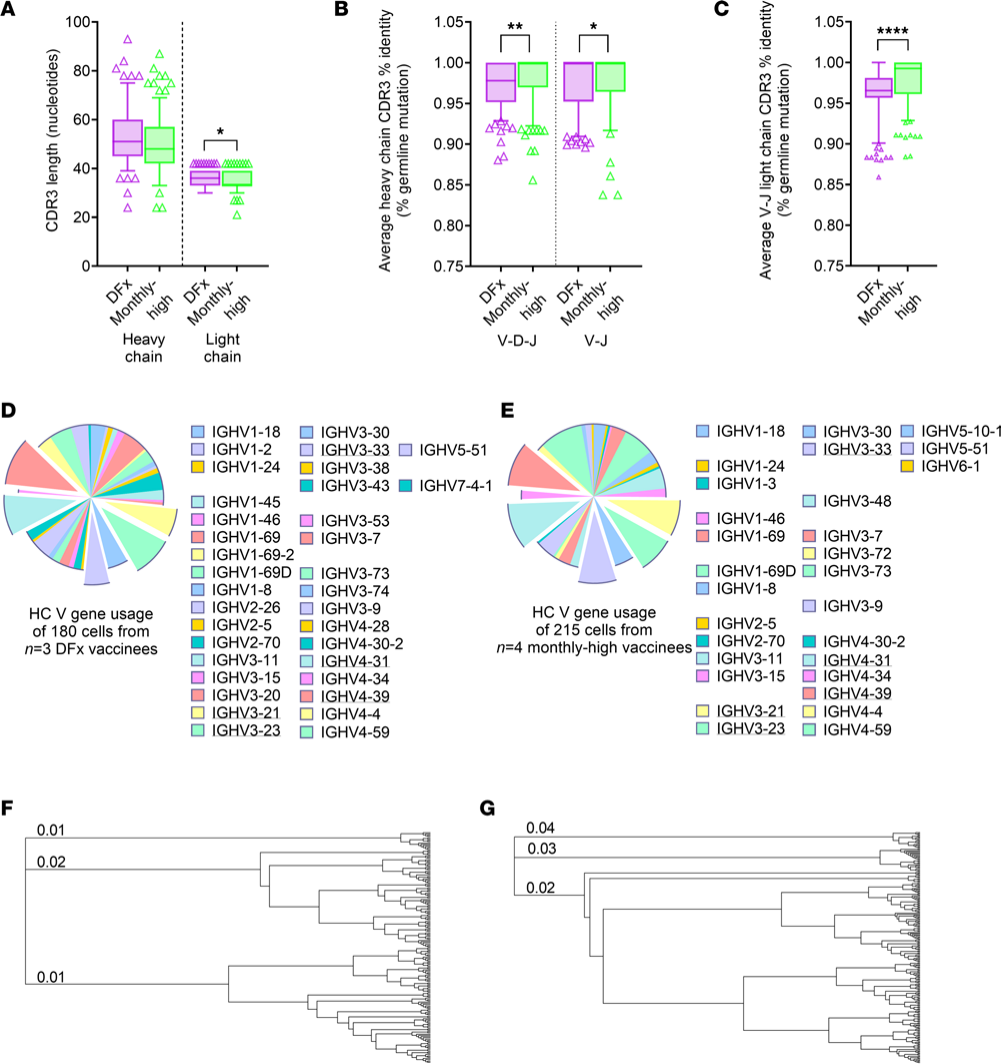
CDR3 sequence analysis of PfRH5-specific B cells in DFx and monthly-high dosing vaccinees. PBMC from prevaccination (Pre-vacc.), and 2-weeks post final vaccination in DFx vaccinees (*n* = 3) and monthly-high vaccinees (*n* = 4) were enriched for B cells and then stained with phenotypic markers for single cell sorting of antigen-specific B cells as defined as: live CD19^+^IgG^+^ lymphocytes that costained for monobiotinylated PfRH5-PE and monobiotinylated PfRH5-APC (gating strategy shown in Supplemental Figure 1A). Libraries were sequenced following a Smart-Seq v4 and Nextera XT pipeline on a HiSeq4000. (**A**–**C**) CDR3 sequences were extracted using the MiXCR pipeline to compare heavy and light chain CDR3 lengths (**A**), and average percentage germline identity for V-D-J or V-J heavy chain (**B**), and V-J light chain (**C**) sequences. Comparisons were performed by Mann-Whitney *U* tests. Central box lines indicate medians, and whiskers denote 5th and 95th percentiles; samples outside the 5th–95th percentile range are shown as triangles. *P < 0.05, **P < 0.01, ****P < 0.0001. (**D** and **E**) Heavy chain (HC) V gene usage is reported for DFx (**D**) and monthly-high (**E**) vaccinees. The top 5 HC V genes shared between groups (IGHV3-21, IGHV3-23, IGHV3-33, IGHV4-31, and IGHV4-39) are emphasized in the charts and underlined in the key. (**F** and **G**) Hierarchical clustering was performed on CDR3 HC amino acid sequences and visualized as dendrograms for DFx vaccinees (**F**) and monthly-high vaccinees (**G**). The first 3 branches are labeled in each dendrogram with branch length (distance between internal nodes) — i.e., substitutions per amino acid.

We also interrogated the BCR repertoire of the circulating PfRH5-specific B cells to determine if there were differences in percentage germline mutation, given the higher avidity in the DFx vaccinee polyclonal anti–PfRH5 IgG (Figure 3; ref. 1). Following analysis with the MiXCR pipeline to extract CDR3 sequences from the scRNA-Seq data set, we compared CDR3 length as well as heavy and light chain CDR3 V-(D)-J percentage germline identity between monthly-high and DFx vaccinees. Here, we observed minimal differences in CDR3 length (Figure 5A), but we observed increased somatic hypermutation in the DFx regimen PfRH5-specific cells as compared with those from the monthly-high regimen (Figure 5, B and C). Analysis of heavy chain V gene usage showed substantial variation between individuals (Supplemental Figure 5) but broadly similar patterns when comparing monthly-high regimen and DFx vaccinee groups (Figure 5, D and E). A total of 40 V genes were detected in CDR3 heavy chains, with 5 of the 6 top genes the same in both monthly-high and DFx vaccinees (IGHV3-21, IGHV3-23, IGHV3-33, IGHV4-31, and IGHV4-39). Finally, hierarchical clustering was performed with heavy chain CDR3 amino acid sequences using Geneious Tree Builder and was visualized as unrooted dendrograms (Figure 5, F and G, and Supplemental Figure 5). Three clusters were observed in both monthly-high regimen (Figure 5F) and DFx vaccinees (Figure 5G) as well as individual vaccinees (Supplemental Figure 5).

## Discussion

The capacity of human vaccine-induced antibodies to neutralize a blood-stage malaria infection (as measured in vitro or in vivo) is strongly linked to the concentration of antibodies present at the time of infection (1). Understanding how to improve the durability of vaccine-specific serum antibody is, therefore, central to the development of a vaccine with long-lasting protective efficacy. In this study, we have interrogated the circulating antigen-specific Ig and B cell responses following immunization with either monthly (0-1-2 month) or DFx (0-1-6 month) dosing regimens using the same vaccine. To the best of our knowledge, this is the first detailed exploration of B cell and humoral responses to a vaccine regimen that significantly improves Ig durability in humans.

We observe that the DFx regimen greatly enhances the longevity of the IgG1 response, with additional indications of improved serum maintenance across multiple other subclasses and isotypes. While the serum antibody kinetics are unique to DFx, analysis of peak Ig responses suggested some similarities with the monthly-high regimen group with the same “high” (50 μg) first and second doses. For example, these 2 groups shared increased IgG4 titers and decreased ADCD, in comparison with the monthly-low and monthly-medium vaccinees. The prevailing view of chronic exposure to antigen as a driver of IgG4 (reviewed in refs. 33, 34) seems consistent with this increased IgG4 in groups primed with the highest doses of antigen. Elevated antigen-specific IgG4 following delayed dosing has also been observed previously with the PfCSP-based preerythrocytic malaria vaccine RTS,S/AS01 (0-1-7 month in this instance; Fx017M) when compared with a monthly 0-1-2 month regimen, as well as an IgG4-related decrease in ADCP (35). Further investigation of the quality of the Tfh cell response may be informative to understand these differences in IgG subclass, and indeed we have previously reported a higher frequency of Tfh2 cells within the PfRH5-specific Tfh cell population in DFx as compared with monthly vaccinees (1).

The significance of dosing regimen–mediated changes to Ig subclass and Fc-mediated functionality of vaccine-specific antibody will clearly vary by antigen and pathogen. For example, while anti-PfRH5 ADCD was not associated with in vivo parasite growth inhibition following blood stage *P*. *falciparum* controlled human malaria infection (CHMI) in our previously published systems serology analyses (1), the role of complement in protection from bacterial infections is well established. DFx dosing would consequently only be appealing for vaccine development programs against bacteria such as *Shigella*, *Neisseria meningitidis*, and *Bordetella pertussis* if vaccine-specific antibody ADCD functionality was retained alongside improvements to serum IgG durability (36–38); in this case, priming with relatively low doses of antigen could be useful in skewing toward this antibody-mediated function. With respect to PfRH5 vaccine development, recovering ADNP functionality with modified DFx regimens may be more important, given its correlation with in vitro parasite growth inhibition (1). Although reduced ADNP was not identified as part of the feature set discriminating between DFx and monthly regimen vaccinees, we did observe a decrease as compared with monthly-medium vaccinees in the univariate analyses. The concept of a potential detrimental effect of excessive antigen or antigen saturation on vaccine immunogenicity has previously been proposed by others and, thus, merits further investigation (9, 39). This could include assessment of DFx regimens that use lower priming doses of antigen. For example, Fc-mediated functionalities of PfRH5-specific antibody could be recovered with a 10 µg/10 µg/2 µg dosing regimen at 0-1-6 months. In short, our data suggest a potentially negative impact of priming with “high” first and second doses of antigen (e.g., reduced capacity to induce Fc-mediated innate pathways) and a positive impact of the delayed fractional boost dose (e.g. increased avidity and FcRn binding) on the PfRH5-specific Ig response with the current DFx regimen. However, optimization of vaccine dose and regimen will depend on the antigen/pathogen in question and the desired antibody function outcomes that mediate protection.

The effect of improved avidity in the specific context of PfRH5-based vaccines is, however, uncertain. Previous work with mAbs (derived from samples from the viral vector trial) indicated that the speed of antibody binding, rather than avidity, is more relevant for in vitro antiparasitic functionality (1, 40). However, improved antibody avidity is associated with increased protection against other pathogens (including preerythrocytic *P*. *falciparum* malaria; refs. 7, 41–44); thus, these DFx data may be of great utility to other vaccine development programs. Further studies are also underway to interrogate the binding kinetics of mAbs derived from monthly regimen and DFx RH5.1/AS01_B_ vaccinees and the relationship to the in vitro GIA functional correlate of protection (1).

In terms of enhanced FcRn binding, the biological implication here relates to the central role of FcRn binding in antibody longevity through promoting recycling rather than lysosomal degradation of serum IgG. Indeed, durability of mAb-biologics can be enhanced by modifying the Fc to improve FcRn binding (45, 46). FcRn binding can also be ameliorated with more highly galactosylated Fc glycans (14), and consistent with this, the systems serology signature associated with the DFx samples — as compared with monthly-high vaccinees — included increased proportions of 2 bigalactosylated glycan moieties (G2B and G2S2F). Evidence from influenza vaccine antibody analyses demonstrating increases in sialylation (e.g., as in G2S2F) correlated with generation of higher affinity antibodies (14, 47). Therefore, it is possible that, in DFx vaccinees, there is an increase in expression of the glycosyltransferases responsible for adding galactose (B4GALT1) and sialic acid (ST6GAL1) with functional significance for FcRn binding/longevity (galactose) and avidity (sialic acid). These hypotheses require further interrogation, however — especially given that the glycosylation changes are more specific than increases in all bigalactosylated or bisialylated moieties. To note, although adjuvant selection can demonstrably impact peak antibody responses and antibody quality (including Fc glycans), evidence of any effect on antibody long-term maintenance is, at present, limited to nonhuman primates (48, 49).

These changes in antigen-specific Ig quantity and quality, thus, suggest fundamentally different B cell responses following the third dose in DFx versus monthly regimens. Our B cell flow cytometry and scRNA-Seq data point to an increase in magnitude of the vaccine-specific response with DFx dosing, as well as a decrease in proportion of plasma cells. At first glance, these results are surprising, given anti-PfRH5 serum IgG levels are comparable between the 2 regimens at the peak time point used for this analysis and are subsequently higher in DFx vaccinees long-term (1). However, LLPCs exit later than mBCs in germinal center development, and while LLPC egress has been documented from 2 weeks after vaccination onward, it is possible that our scRNA-Seq time point is better suited to mBC and short-lived plasma cell (SLPC) detection as compared with (later) LLPCs (50–52). Accordingly, we propose that the 2-week post–final vaccination plasma cell signal in monthly-high vaccinees is derived predominantly from mBCs that have differentiated into SLPCs following antigen reexposure, rather than those that have returned to a draining lymph node and differentiated into LLPCs via germinal center reactions. It is also likely that monthly regimen vaccinees still possessed ongoing PfRH5-specific germinal center reactions from the second vaccination at the time of the final booster, which — alongside higher concentrations of anti-PfRH5 serum IgG at the time of vaccination — could have dampened new germinal center responses (9, 53, 54). This is consistent with HIV vaccinology data from nonhuman primates, where longer intervals between doses were associated with increased germinal center B cell responses (55). We accordingly suspect that the major impact on magnitude of germinal center development comes from the delay of the final booster. To note, others have speculated that the improved avidity with DFx is driven by greater germinal center competition for antigen due to the fractionation of the final dose rather than the delay (7, 39, 56, 57). These hypotheses are not necessarily contradictory; modeling data indicate that IgG concentration and avidity may be regulated independently (58). Delineation of the effects of delay versus fractionation is a focus of an ongoing PfRH5 Phase 1b trial in Tanzania (NCT04318002).

Future clinical trials should, therefore, seek to more precisely define plasma cell population kinetics with DFx versus monthly boosting and to confirm our hypothesized skew toward LLPC and SLPC subsets, respectively. This distinction is currently difficult, given the lack of clear markers to resolve SLPCs from LLPCs/LLPC precursors in humans (reviewed in ref. 59), although data suggest that the transcription factor Zbtb20 may be used to define LLPCs in mice (60); however, no increased expression was observed in our putative SLPC population. Analyses moving forward will, thus, rely on more frequent venous sampling following the final vaccination, ideally coupled with larger sample sizes and analyses of draining lymph node aspirates to directly monitor germinal center formation/longevity and define peripheral biomarkers of plasma cell output (54, 61, 62). Given the relatively small sample size of our scRNA-Seq analyses, recapitulating our findings with the same dosing regimen in additional vaccinees may also be worthwhile. BM aspirates would also be of use to confirm a link between higher serum anti-RH5.1 IgG maintenance and presumed higher LLPC seeding in the DFx regimen, as well as with putative LLPC precursor populations.

Finally, it will be of great interest to better understand the discrepancy between the results observed with DFx RH5.1/AS01_B_ vaccination and similar Fx017M dosing with the RTS,S/AS01, which shows indications of greater protection than the monthly regimen in CHMI studies (6–8, 39). Like with DFx RH5.1 dosing, the Fx017M RTS,S regimen increased IgG avidity and (bulk plasmablast — i.e., SLPC) B cell somatic hypermutation after final vaccination. This correlated with protection but not magnitude of the IgG response as compared with the monthly 0-1-2 month regimen (7). PfCSP-specific mBCs were also indirectly detected at higher frequencies in Fx017M vaccinees as compared with the monthly 0-1-2 month regimen following 5-day PfCSP stimulation (9). However, unlike with RH5.1/AS01_B_, there was no apparent Fx017M-mediated benefit to Ig longevity and, thus, durability of vaccine-mediated protection (7). This is a critical distinction to understand in order to ensure relevance of DFx dosing to other antigens and pathogens. One possibility is that the failure of Fx017M to improve Ig serum maintenance is related to the valency of the antigen; PfCSP is a repeating antigen arrayed on a virus-like particle (VLP) in RTS,S that could form immune complexes affecting LLPC development (63). Other discrepancies are also found with recently published system serology analyses of monthly versus Fx017M dosing (64) where trends were observed with Fx017M different from those reported here with DFx.

In conclusion, the data presented here support the DFx regimen as a promising dosing schedule for optimizing the humoral response against difficult pathogens like blood-stage malaria that require high, sustained titers for protection. The impact appears to be largely related to IgG1, but more subtle effects on other isotypes and subclasses are also present. Two hypotheses regarding the underlying mechanism of the improved serum antibody longevity in the DFx schedule merit further exploration: increased recycling through enhanced FcRn binding and a potential shift in B cell fate from SLPC to germinal center–derived LLPC following the delayed final dose. Further clinical trials will be needed to directly compare the impact of delayed boosting to the fractionation of the final dose and also — for antigens other than PfRH5, such as PfCSP — to delineate the possible roles of antigen and vaccine delivery platform. With respect to platform, it will be of particular interest to determine whether delayed booster dosing with mRNA vaccines can generate a comparable impact on vaccine-specific B cell and Ig responses. Data from SARS-CoV-2 mRNA vaccine trials have shown enhanced peak antibody responses with longer intervals between the prime and boost vaccinations but, as of yet, no significant impact on serum antibody durability (3). While it is possible that this reflects a difference between humoral responses to protein versus mRNA antigen delivery, it will be key to investigate longer booster dosing delays in the context of mRNA vaccination. Understanding how vaccine antigen, dose, regimen, and delivery platform interact to shape the humoral immune response in humans, thus, remains a key challenge for the future.

## Methods

### Experimental model and subject details.

This study focused on the comparison of immune responses between groups receiving different dosing regimens of PfRH5 protein (RH5.1) with AS01_B_ adjuvant (ClinicalTrials.gov, NCT02927145; adjuvant provided by GSK) (1, 65).

Vaccine regimens are presented in Table 1. Total IgG and Tfh cell responses to the different regimens have already been reported elsewhere (1, 10). Vaccinee age and sex were comparable between regimens and are summarized in Table 1. Not all vaccinees could be run in each of the assays in this study; specific sample sizes are specified in figure legends.

A second PfRH5 clinical trial with heterologous viral vectors (consisting of a ChAd63-PfRH5 prime, followed by an MVA-PfRH5 boost) is also briefly referenced for comparison in Figure 1 (ClinicalTrials.gov, NCT02181088) (10, 11).

### PfRH5-specific B cell flow cytometry.

Cryopreserved PBMC were thawed into R10 media (RPMI [R0883, MilliporeSigma] supplemented with 10% heat-inactivated FCS [60923, Biosera], 100 U/mL penicillin/0.1 mg/mL streptomycin [P0781, MilliporeSigma], 2 mM L-glutamine [G7513, MilliporeSigma]) and were then washed and rested in R10 for 1 hour. B cells were enriched (Human Pan–B cell Enrichment Kit [19554, StemCell Technologies]) and then stained with viability dye FVS780 (565388, BD Biosciences). Next, B cells were stained with anti–human CD19-PE-Cy7 (557835, BD Biosciences), anti–human IgG-BB515 (564581, BD Biosciences), anti–human IgM-BV510 (563113, BD Biosciences), anti–human CD27-BV711 (564893, BD Biosciences), and anti–human CD21-BV421 (562966, BD Biosciences) as well as 2 fluorophore-conjugated PfRH5 probes. Preparation of the PfRH5 probes has been published previously (10). In brief, monobiotinylated PfRH5 was produced by transient cotransfection of HEK293F cells with a plasmid encoding BirA biotin ligase and a plasmid encoding a modified PfRH5. The PfRH5 plasmid was based on ‘RH5-bio’ (a gift from Gavin Wright; University of York, York, United Kingdom; Addgene plasmid 47780; http://n2t.net/addgene:47780;RRID:Addgene_47780; ref. 66). RH5-bio was modified prior to transfection to incorporate a C-tag for subsequent protein purification, as well as a 15–amino acid deletion at a predicted C-terminus cleavage site and a 115–amino acid deletion from the linear N-terminus. Probes were freshly prepared for each experiment, by incubation of monobiotinylated PfRH5 with streptavidin-PE (S866, Invitrogen) or streptavidin-APC (405207, eBioscience) at an approximately 4:1 molar ratio to facilitate tetramer generation and subsequent centrifugation to remove aggregates (13,000–14,000 rpm [max microcentrifuge speed] at room temperature for 10 minutes). Following surface staining, cells were fixed with CytoFix/CytoPerm (554714, BD Biosciences), washed, and stored at 4°C until acquisition.

Memory PfRH5-specific IgG^+^ B cells identified as live CD19^+^CD21^+^CD27^+^IgG^+^IgM^–^ PfRH5/APC^+^PfRH5/PE^+^ lymphocytes (Supplemental Figure 1) and acquired on a Fortessa X20 flow cytometer with FACSDiva8.0 (both from BD Biosciences). Samples were analyzed using FlowJo (v10; Tree Star Inc.). Samples were excluded from analysis if there were < 100 cells in the parent population.

### Standardized ELISAs.

Standardized ELISAs were used to quantify serum RH5.1–specific IgG1, IgG2, IgG3, IgG4, IgA, IgA1, IgA2, and IgM responses in vaccinees. Nunc MaxiSorp flat-bottom ELISA plates (44-2404-21, Invitrogen) were coated overnight with 5 μg/mL of RH5.1 protein in PBS. Plates were washed with washing buffer composed of PBS containing 0.05% TWEEN 20 (P1379, Sigma-Aldrich) and blocked with 100 μL of Blocker Casein in PBS (37582, Thermo Fisher Scientific). After removing blocking buffer, standard curve and internal controls were created in casein using a pool of high-titre volunteer plasma, specific for each isotype or subclass being tested, and 50 μL of each dilution was added to the plate in duplicate. Test samples were diluted in casein to a minimum dilution of 1:50, and 50 μL was added in triplicate. Plates were incubated for 2 hours at 37°C and washed in washing buffer. An alkaline phosphatase–conjugated secondary antibody from Southern Biotech was diluted at the manufacturer’s recommended minimum dilution for ELISA in casein. The antibody used was dependent on the isotype or subclass being assayed and were as follows: mouse anti–human IgG1 Fc-AP (catalog 9054-04), IgG2 Fc-AP (catalog 9060-04), IgG3 hinge-AP (catalog 9210-04), IgG4 Fc-AP (catalog 9200-04), IgA1-AP (catalog 9130-04), and IgA2-AP (catalog 9140-04) and goat anti–human IgA-AP (catalog 2050-04) and IgM-AP (catalog 2020-04). In total, 50 μL of the secondary antibody dilution was added to each well of the plate and incubated for 1 hour at 37°C. Plates were developed using PNPP alkaline phosphatase substrate (N2765, Sigma-Aldrich) for 1–4 hours at 37°C. Optical density at 405 nm was measured using an ELx808 absorbance reader (BioTek) until the internal control reached an OD_405_ of 1. The reciprocal of the internal control dilution giving an OD_405_ of 1 was used to assign an AU value of the standard. Gen5 ELISA software v3.04 (BioTek) was used to convert the OD_405_ of test samples into AU values by interpolating from the linear range of the standard curve fitted to a 4-parameter logistics model. Any samples with an OD_405_ below the linear range of the standard curve at the minimum dilution tested were assigned a minimum AU value according to the lower limit of quantification of the assay.

### GIA assay.

GIA of pre- and postvaccination serum samples at 10 mg/mL total IgG was assessed at the GIA Reference Center (NIAID, NIH) as previously described (1, 10, 11, 67). In brief, purified IgG samples were incubated with *P*. *falciparum*–infected RBCs for 40 hours at 37°C, and biochemical determination of parasite lactate dehydrogenase was used to quantify final parasitemia in each well.

### Systems serology.

The PfRH5 systems serology analyses were performed as previously reported (1). A total of 49 parameters were measured in the following assays: THP-1 phagocytosis, neutrophil phagocytosis, NK cell activation (3 read-outs), complement deposition, antibody isotype and subclass (9 read-outs including total IgG avidity), FcR binding (8 read-outs), C1q binding, and frequencies of glycan structures (13 read-outs).

Details of individual assays are described in the Supplemental Methods.

### scRNA-Seq of PfRH5-specific CD19^+^IgG^+^ B cells.

B cells were enriched (Human Pan–B cell Enrichment Kit [19554, StemCell Technologies]) from cryopreserved PBMC samples from 2 weeks after the final vaccination in *n* = 3 DFx vaccinees and *n* = 4 monthly-high vaccinees. The 2-week postvaccination time point was selected to maximize the number of cells available for sequencing. These samples were then stained with anti–human CD19-PE-Cy7 (557835, BD Biosciences), anti–human IgG-BB515 (catalog 564581), and FVS780 (565388, BD Biosciences) as well as 2 fluorophore-conjugated PfRH5 probes (see above; ref. 10). PfRH5-specific B cells identified as live CD19^+^IgG^+^PfRH5/APC^+^PfRH5/PE^+^ lymphocytes were single-cell sorted on a BD FACS Aria (BD Biosciences) into Buffer TCL (1031576, Qiagen) with 1% 2-mercaptoethanol. Sorted cells were snap frozen on dry ice before storage at –80°C until processing.

mRNA from thawed single B cells was purified with RNAClean XP beads (A63987, Beckamn Coulter) and converted to cDNA using dT_30_VN and TSO oligonucleotides and SMARTScribe reverse transcriptase (639538, Clontech) with a modified Smart-Seq v4 for Ultra Low Input RNA protocol (Takara Bio). Both steps were done in the presence of a recombinant RNase inhibitor (2313B, Takara Bio). cDNA was then amplified with SeqAmp DNA Polymerase (638509, Clontech).

HighPrep PCR beads (AC-60050, MagBio) were used to purify cDNA prior to quantification with Qubit dsDNA HS Assay Kit (Q32854, Invitrogen) and cDNA normalization. Sequencing libraries were created using the Nextera Index Kit v2 (FC-131-2001, Illumina) and the Nextera XT DNA Sample Preparation Kit (FC-131-1096, Illumina). Libraries were then purified with AMPure XP beads (A63881, Beckman Coulter) and quantified by quantitative PCR (qPCR) with Library Quantification Kit - Illumina/ABI Prism (KK4835, KAPA Biosystems). Cells yielding libraries > 1 nM by qPCR were normalized and pooled by vaccinee for sequencing on the HiSeq4000 platform (Illumina) to an average read count of 4,113,948 reads per cell. All cells were sequenced in a single run. Sequencing was performed on a total of 226 cells from monthly-high vaccinees (range 37–88 cells) and 228 cells from DFx vaccinees (range 59–88 cells). Sequencing files are available from the NCBI SRA database, BioProject accession no. PRJNA855352 (https://www.ncbi.nlm.nih.gov/bioproject/?term=PRJNA855352).

### Statistics.

Comparisons were performed between regimens with (2-tailed) Mann-Whitney *U* tests or Kruskal-Wallis with Dunn’s correction for multiple comparisons. *P* < 0.05 was considered statistically significant. Statistical analyses for systems serology and scRNA-Seq data are outlined below. For all data, relevant statistical tests are specified in figure legends.

Regarding computational analysis of systems serology data, multivariate analysis of the systems serology data was performed in R with the following approach. Features with missing measurements for more than 50% of subjects were removed from this analysis. Missing values were then imputed using k-nearest neighbors (k = 3, R package “DMwR” v.0.4.1), and all data were mean-centered and variance-scaled (*Z* scored). Univariate differences were assessed with Kruskal-Wallis tests, using the Benjamini-Hochberg multiple hypothesis correction. Significant differences were then assessed in a pairwise manner with Mann-Whitney *U* tests. PLS-DA was performed using the “ropls” (v.1.22.0) and “systemsseRology” (v1.0) packages of R for model building and cross-validation/visualization, respectively. Significant features were chosen via the LASSO feature selection algorithm, which was run 100 times on the entire data set using the function “select_lasso” from the “systemsseRology” R package (v1.0). Features chosen in at least 80% of repetitions were used to build PLS-DA classifiers. PLS-DA model performances were then assessed using a 5-fold cross-validation approach, and reported cross-validation accuracy is the mean of 10 rounds of 5-fold cross-validation, which includes 100 repeats of feature selection per fold, per round. To assess the importance of selected features, negative control models were built both by permuting group labels and by selecting random, size-matched features in place of true selected features. Ten rounds of cross-validation with 5 permutation and 5 random-feature trials per round were performed (again with 100 repeats of feature selection per fold per round for the permutation trials), and exact *P* values were obtained from the tail probability of the generated null distribution. Correlation networks were built to reveal additional serology features significantly associated with the selected features. Serology features significantly (*P* < 0.05, after a Benjamini-Hochberg correction) correlated (Spearman *r*_s_ > |0.7|) via Spearman correlation were selected as cocorrelates. Correlation coefficients were calculated using the “correlate” function in the “Corrr” package (v0.4.3), with *P* values corrected using “p.adjust” from the “stats” package (v4.0.3). Network visualization was performed using “ggraph” (v2.0.5) and “igraph” (v1.2.6) packages, with manual label and node positioning corrections made in Adobe Illustrator (v2020) for improved visualization. The gradient color of edges represents correlation values between the features, represented as nodes. Nodes are colored according to selected status, with gray nodes as selected features and white nodes as cocorrelate features. An R notebook for the analysis is available.

scRNA-Seq gene expression analysis was performed using the Human Cell Atlas instance of the Galaxy biocomputing framework (https://humancellatlas.usegalaxy.eu; refs. 68, 69) based on the “Reference-based RNA-Seq data analysis” (70) and “Preprocessing of Single-Cell RNA data” (71, 72) workflow templates. Paired-end FASTQ reads were aligned to the human genome (hg19) with gene annotations from Ensembl (Homo_sapiens.GRCh37.75.gtf; ref. 73) using Trimmomatic (74), followed by RNAStar (75). After removing reads mapped multiple times, the number of reads mapped to each gene was quantified with FeatureCounts (76) using a GTF file groomed with StringTie Merge (77) to annotate genes. Heterogeneity in gene expression was then explored with either Seurat (78) in R (v4.1.0) — including PCA for downstream Louvain clustering and UMAP visualization — or with DESeq2 and Annotate DESeq2 in Galaxy. Harmony (79) was used to adjust for the possibility of batch effects prior to the clustering/UMAP analyses. Expression of genes of interest was compared between cluster 4 and other clusters using Wilcoxon rank sum tests, with Bonferroni correction for multiplicity of testing (individual genes; *P*_adj_). Enrichment of genes in a plasma cell gene set (“TARTE_PLASMA_CELLS_VS_B_LYMPHOCYTE_UP” accessed from https://www.gsea-msigdb.org/gsea/msigdb; ref. 30) was likewise compared with a Kruskal-Wallis test (gene set; *P* value). Differentially expressed genes identified with DESeq2 are reported alongside adjusted *P* values (*P*_adj_) following an FDR correction (Benjamini-Hochberg). Pathway analysis was also performed using Fgsea (fast GSEA; ref. 80) using either all transcript counts or only those with significantly different expression between groups (*P*_adj_ < 0.05 following DESeq2). Hallmark and KEGG mSigDB gene sets were used to define enriched gene sets from previously curated databases.

BCR CDR3 repertoire analysis was performed with the MiXCR Analyze shotgun pipeline, designed for clonotype analysis of non–(VDJ)-enriched RNA-Seq data (81, 82). Only productive rearrangements were considered for downstream analysis. If multiple heavy or light chain clones were reported for a given cell, the clones with the highest read counts were used for analysis. CDR3 percentage germline identities were calculated as averages from V-D-J (heavy chain clones only) or V-J only (heavy and light chain clones). CDR3 length and V gene usage are direct outputs of MiXCR. Hierarchical clustering of CDR3 amino acid sequences was performed using Geneious Tree Builder (Alignment type: Global alignment; Genetic Distance Model: Jukes-Cantor; Tree Build Method: Neighbor-Joining). Dendrograms were generated in Geneious (showing unrooted tree as rooted; proportional transformation).

### Study approval.

The clinical trial NCT02927145 from which samples were used for this study was approved by the Oxford Research Ethics Committee A in the UK (REC reference 16/SC/0345) as well as by the UK Medicines and Healthcare products Regulatory Agency (MHRA; reference 21584/0362/001-0001). The NCT02181088 trial was also approved by the Oxford Research Ethics Committee A in the UK (REC reference 14/SC/0120) as well as by the UK MHRA (reference 21584/0331/001-0001). All volunteers gave written informed consent. These assays were performed at MGH using plasma samples from the RH5.1 protein/AS01_B_ trial (1) and were deemed not human research following review by the MGH IRB (protocol no. 2012P002452). Additionally, human whole blood and buffy coats were collected at MGH from healthy donors who did not participate in the RH5.1 protein/AS01_B_ trial. Use of these internal samples as sources of uninfected primary neutrophils and NK cells was deemed not human research by the MGH IRB (protocol nos. 2010 P002121 and 2005 P001218).

## Author contributions

CMN led the study. ROP, AMM, and SJD were chief (AMM), principal (ROP), or lead (SD) investigators on the clinical trials. CMN, JRB, JKF, ARM, FL, AD, KM, and SES performed experiments. CMN, JRB, CD, JKF, C. Goh, C. Griffin, AK, CL, SD, MT, AD, KM, CAL, DAL, and GA analyzed and/or reviewed data. FL, JRF, and AR supported project management and training. CAL, ROP, AMM, RAS, DCD, GA, and SJD contributed reagents, materials, and/or analysis tools. CMN wrote the manuscript.

## Supplementary Material

Supplemental data

## Figures and Tables

**Table 1:**
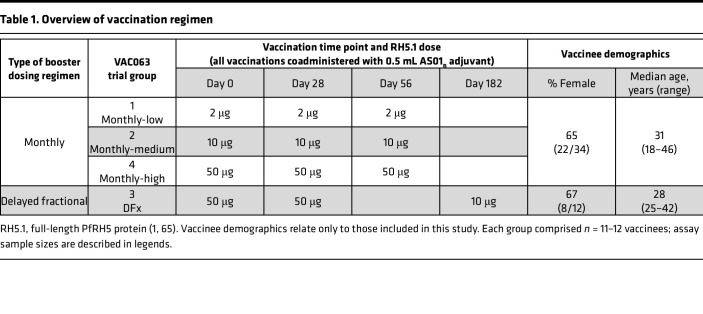
Overview of vaccination regimen

**Table 3:**
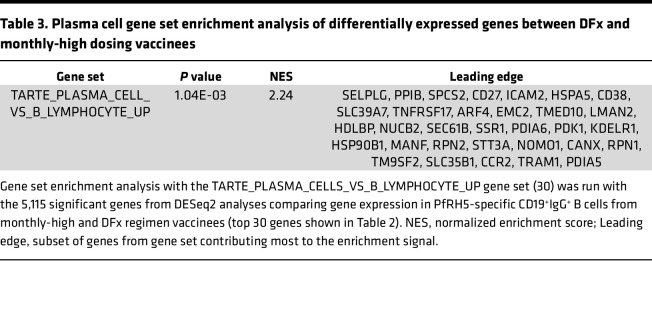
Plasma cell gene set enrichment analysis of differentially expressed genes between DFx and monthly-high dosing vaccinees

**Table 2:**
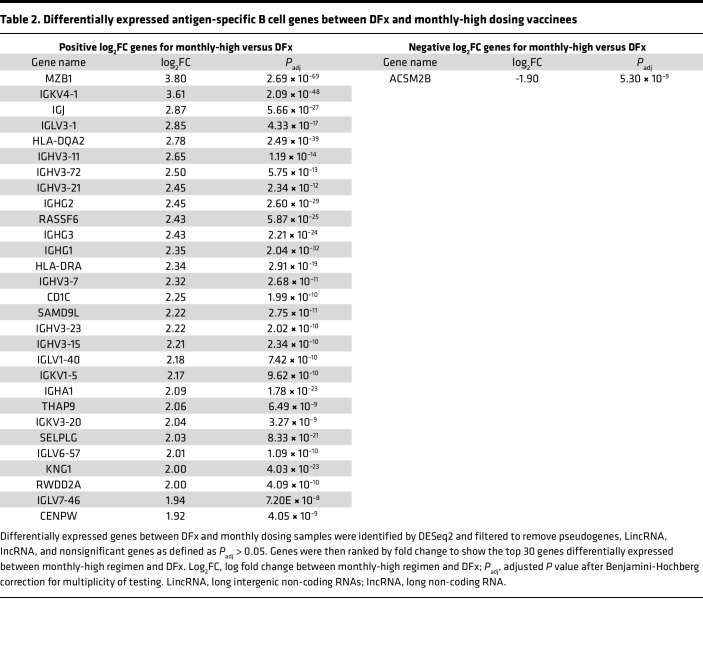
Differentially expressed antigen-specific B cell genes between DFx and monthly-high dosing vaccinees

## Version 1 01/24/2023

Electronic publication
